# Sexual Conspecific Aggressive Response (SCAR): A Model of Sexual Trauma that Disrupts Maternal Learning and Plasticity in the Female Brain

**DOI:** 10.1038/srep18960

**Published:** 2016-01-25

**Authors:** Tracey J. Shors, Krishna Tobόn, Gina DiFeo, Demetrius M. Durham, Han Yan M. Chang

**Affiliations:** 1 Behavioral and Systems Neuroscience, Department of Psychology, Center for Collaborative Neuroscience, Rutgers University.

## Abstract

Sexual aggression can disrupt processes related to learning as females emerge from puberty into young adulthood. To model these experiences in laboratory studies, we developed SCAR, which stands for Sexual Conspecific Aggressive Response. During puberty, a rodent female is paired daily for 30-min with a sexually-experienced adult male. During the SCAR experience, the male tracks the anogenital region of the female as she escapes from pins. Concentrations of the stress hormone corticosterone were significantly elevated during and after the experience. Moreover, females that were exposed to the adult male throughout puberty did not perform well during training with an associative learning task nor did they learn well to express maternal behaviors during maternal sensitization. Most females that were exposed to the adult male did not learn to care for offspring over the course of 17 days. Finally, females that did not express maternal behaviors retained fewer newly-generated cells in their hippocampus whereas those that did express maternal behaviors retained more cells, most of which would differentiate into neurons within weeks. Together these data support SCAR as a useful laboratory model for studying the potential consequences of sexual aggression and trauma for the female brain during puberty and young adulthood.

Thirty percent of women worldwide experience some kind of physical or sexual violence in their lifetime^1^, and adolescent girls are much more likely than the general population to be victims of rape, attempted rape, or sexual assault^2^. Nearly one of every four undergraduate women experience sexual aggression and violence while at university, most occurring in freshmen and sophomores^3^. Moreover, individuals with mental illness, especially those who are poor and homeless, are especially susceptible to sexual aggression and violence while living on the streets^4^,^5^. Regardless of when or where, sexual aggression and abuse is one of the most stressful and traumatic of life experiences, often contributing to the emergence of negative affect, anxiety, deficits in learning and depression in adulthood^6^,^7^,^8^. Despite the undeniable connection between sexual trauma in women and mental health disorders, we know little about how sexual aggression and related experiences alters the female brain. One of the reasons is because there is no established animal model for studying the consequences of sexual trauma on behavior and neuronal function in females.

Most models of stress in laboratory studies rely on exposure to restraint stress, swim stress or aversive shocks, which do not necessarily reflect the kinds and types of stressors that young women experience in real life. Nonetheless, using these and similar models, we have published numerous studies indicating that female rodents respond very differently than male rodents do to laboratory stressors^9^. For example, associative learning of a classically-conditioned anticipatory response is enhanced after exposure to a laboratory stressor in male rodents but severely compromised in females^10^,^11^. These learning deficits in females were accompanied by decreases in the density of synaptic spines in the hippocampus. The learning deficits in females as a consequence of stress depend on neuronal activity in a number of brain regions, most notable the hippocampus, amygdala, and prelimbic region of the prefrontal cortex^12^,^13^.

It is often assumed that the effects of stress learning and neuronal function in laboratory animals reflect changes that could occur in women who experience stressful life events. One experience that happens often to women and females of many species is sexual aggression, and as noted, these aversive experiences in women can lead to mental health complications as well as distracting thoughts and ruminations about the past that prevent their abilities to learn and concentrate. Even for women who do not go on to develop mental illness, sexually-traumatic experiences leave a lasting impression on their lives, presumably through changes in neuronal processes related to learning and memory. If we are to fully understand the necessary and sufficient neuronal and behavioral mechanisms that are activated within the female brain during sexual aggression, we must develop a laboratory model. To meet this need, we developed an animal model known hereafter as Sexual Conspecific Aggressive Response (SCAR). In the SCAR model, we focused on the female as she transitions from puberty into young adulthood because this is the time period when females are most likely to encounter sexually aggressive adult males. We also chose this time period for practical reasons; the pubescent female rat is not fully capable of copulation and/or reproduction because the vaginal canal is not fully open and/or the estrous cycle is not fully developed. Therefore, interactions with an adult male will not produce offspring. To mimic a novel encounter with an adult male, a pubescent female Sprague Dawley rat (postnatal day 35) was exposed to a sexually-experienced adult male rat for 30-min in a context different from either of their home cages. The encounters were video-recorded in order to score behaviors related to aggression and reception. The adult males were not selected for aggression but rather were sexually-experienced breeders from an established colony. During the experiments, the young female was exposed to two different adult males, one at a time, alternated every other day, throughout puberty.

In the following experiments, we describe the behaviors that occurred during the interactions and report consequences of those interactions. For these initial studies, we focused on the physiological stress response because it is important to establish that the experience is stressful for the female rodent. Concentrations of the stress hormone, corticosterone were measured because its elevation indicates activation of the hypothalamic-pituitary adrenal (HPA) axis, the primary stress response in mammalian species. We next examined the effects of the SCAR experience on learning. We chose the classically-conditioned eyeblink response because exposure to standard laboratory stressors disrupts this type of learning in adult females, as noted above. We also chose this task because this type of learning is likewise disrupted by exposure to an adult male^14^. Therefore, if the SCAR experience were to disrupt learning of this response, one could conclude that social interaction with the male induces similar responses to a more typical laboratory stressor (swim stress, tail stimulation) and also that the effect can extend from puberty into adulthood. In an additional set of experiments, we examined the consequences of the social interaction on the expression of maternal behavior in the female. The development and “learning” of maternal caring behaviors are arguably some of the most if not the most important functions that females acquire. Again, the goal was to evaluate potential outcomes that have direct relevance to behaviors that are meaningful for women but also impact survival of most species.

As a final dependent measure, we considered the potential effects of the SCAR experience on neurogenesis in the hippocampus. The hippocampus generates new neurons throughout life – thousands each day and nearly twice as many during puberty^15^. Many of these new neurons die within a few weeks of being generated unless a new learning experience occurs^16^,^17^. The types of learning that keep new neurons alive include trace conditioning, spatial navigation learning and motor skill learning^17^,^18^,^19^. The effects of learning on cell survival in puberty are similar to those in adulthood but because so many more cells are generated, the consequences of learning (or not learning) for brain integrity are especially profound. In the present experiments, we hypothesized that the effects of SCAR on the expression of maternal behavior would disrupt the survival of newly-generated cells in the hippocampus. The goal was to establish an outcome measure in the female brain that ultimately is affected by repeated encounter with the adult male.

## Methods

Male and female Sprague-Dawley rats were bred at Rutgers University in the Departmet of Psychology. Twenty-eight days after birth, animals were weaned and housed in goups of 2–3 males and 2–4 females in standard plastic shoebox style cages (44.5 cm long by 21.59 cm wide by 23.32 cm high). Female in the maternal study were housed alone. Animals were given access to food and water *ad libitum* and maintained on a 12:12 hr light-dark cycle; the light cycle began at 7am and ended at 7pm. All handling and experimental manipulations were carried out in the light portion of the diurnal cycle. Experiments were conducted with full compliance with the rules and regulation specified by the PHC Policy on Human Care and Use of Laboratory Animals and the Guide for the Care and Use of Laboratory Animals. The Rutgers University Animal Care and Facilities Committee approved all procedures.

### Experiment 1: What behaviors are expressed during SCAR?

SCAR exposures began when the pubescent female was post-natal day (PND) 35, whereas male breeders varied in age from approximately 120–160 days old. The females in this age range weighed between 120–220-g, whereas the males weighed between 400–700-g. During the experimental manipulation, one pubescent female rat (n = 10) was placed in a novel cage with an adult sexually-experienced male rat for 30-min. The behaviors during the pairing were compared to behaviors during a similar pairing between a pubescent female rat (n = 10) and an adult female rat. All the conditions were the same, regardless of the individual pairings. The exposures occurred each day for eight consecutive days. The pubescent female was exposed to one of two adults that were alternated each day. All interactions were video recorded and behaviors were hand scored by two independent experimenters.

Very few sexual intromissions occurred and therefore, data are not presented here. We counted and analyzed three behaviors as follows: 1) anogenital trackings, 2) pins, and 3) escapes. During an anogenital tracking event, the male tracked while presumably sniffing the anogenital region of the female as she ran around the cage. When the snout of the male was touching or nearly touching the anogenital region of the female for a continuous amount of time (>1-sec), we considered this one tracking behavior. During a pin, the adult male would effectively restrain the female, usually by sitting on top of her or turning her over on her back and using his paws to hold her down. During an escape behavior, the female sat up on her rear paws and reached for the top of the cage, as if trying to escape. These three behaviors were counted across the 30-min encounter in 10-min intervals. As noted, these behaviors were compared to the same behaviors expressed by a pubescent female when paired with an adult female (female/female).

### Results Experiment 1

**Figure 1:**
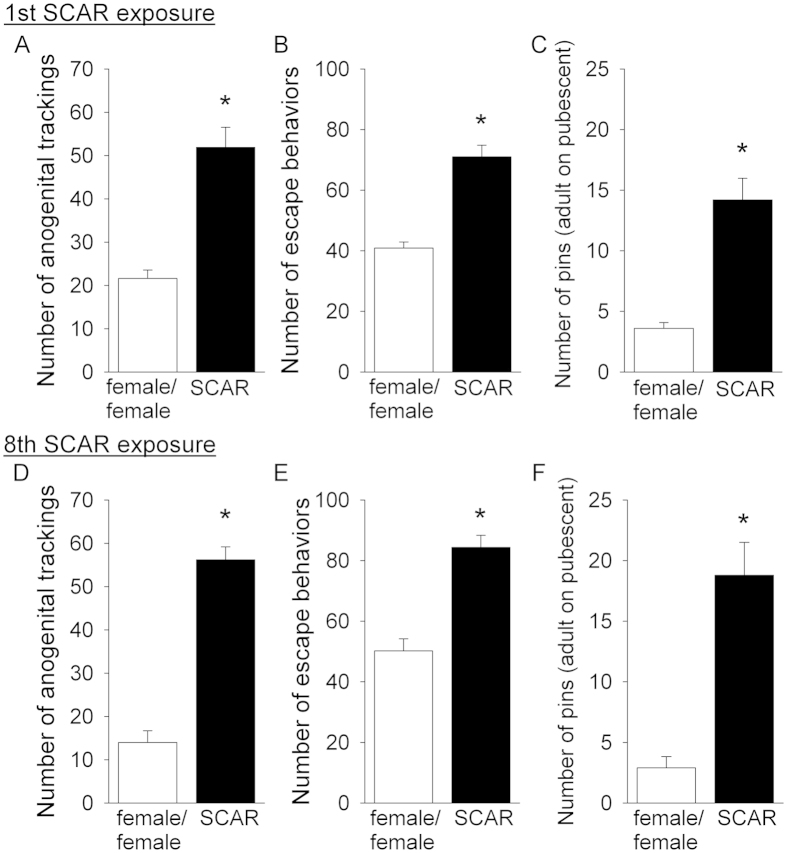
Behavioral measures of SCAR exposures. (**A**) During the first SCAR exposure, the number of anogenital sniffs was significantly greater in the SCAR (adult male/pubescent female) group than in females paired with another female (female/female). (**B**) During the first exposure, the female made a greater number of escape behaviors when paired with an adult male than when paired with an adult female. (**C**) The adult male also pinned the pubescent female down more times than did the adult female.(**D**–**F**) These behavioral results were similar during the eighth exposure. The SCAR group received more anogenital sniffs, emitted more escape behaviors and pins when compared to similar behaviors expressed when a pubescent was paired with an adult female.

During the first SCAR exposure, numbers of anogenital trackings expressed by the adult male (adult male/pubescent female; SCAR) were significantly greater when compared to similar behaviors expressed by an adult female rat paired with a pubescent female (female/female) group (*t*_(18)_ =6.07; *p* < 0.001; Fig. 1A). Numbers of escape behaviors expressed by the pubescent female were also greater in number during the interaction with the adult male than the adult female (*t*_(18)_ = 6.94; *p* < 0.001; Fig. 1B). Numbers of pins were greater in number when the pubescent female was interacting with an adult male than when interacting with an adult female (*t*_(18)_ = 5.77, *p* < 0.001; Fig. 1C). These same behaviors were analyzed during the 8^th^ consecutive day of conspecific exposures. As during the first exposure, numbers of anogenital trackings were elevated (*t*_(18)_ = 10.51; *p* < 0.001; Fig. 1D), as were escape behaviors (*t*_(18)_ = 6.09; *p* < 0.001; Fig. 1E), and numbers of pins (*t*_(18)_ = 5.57; *p* < 0.001; Fig. 1F). Numbers of these behaviors did not change between the first and eighth exposures (p > 0.05). These results suggest that the recorded behaviors did not habituate with continued social interactions between the two conspecifics.

### Experiment 2: Does the SCAR exposure increase corticosterone?

In a second experiment, we analyzed the effects of SCAR exposure on the concentrations of the stress hormone corticosterone at two time points. First, we compared the amount of corticosterone released within the pubescent female 30-min after the exposure to an adult male versus exposure to an adult female. Pubescent females were exposed to either an adult male breeder (n = 6) or an adult female (n = 5, PND 60–120) for 30-min and following the single exposure, trunk blood was collected 30-min later. Animals were given a lethal dose of pentobarbital intraperitoneal injection and trunk blood was collected. Blood was transferred into heparin tubes (BD Biosciences, Franklin Lakes, NJ), centrifuged at 2500 RPM for 20-min and stored at −20 °C. Corticosterone immunoassay was performed according to manufacturer’s protocol (Corticosterone EIA Kit, Arbor Assays, Ann Arbor, MI). In separate groups, a pubescent female was exposed to an adult male for 30-min (n = 8) or was placed alone in a novel cage for 30-min (n = 7). The concentration of corticosterone in the blood of the pubescent female exposed to the adult male was compared to the amount released in response to a novel context, which is mildly stressful for a rodent. Two hours after the interaction had ceased, females were given a lethal dose of pentobarbital as above and blood was collected for radioimmunoassay of corticosterone concentrations.

#### Results Experiment 2

**Figure 2:**
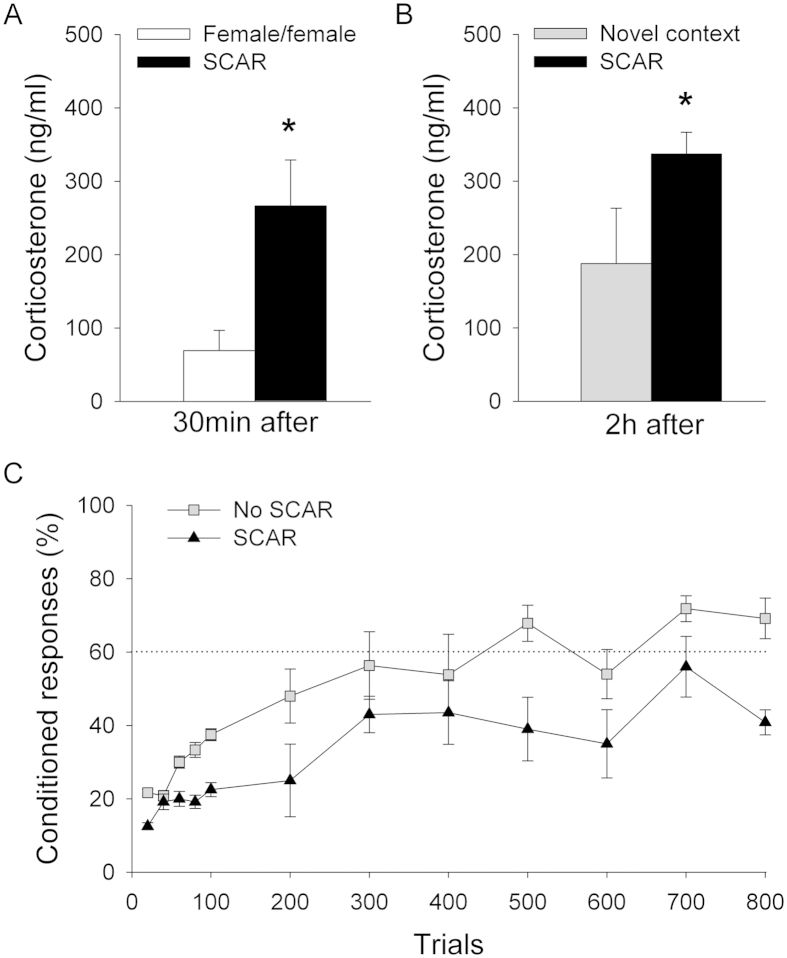
SCAR increases stress hormones and disrupts learning. (**A**) Corticosterone concentrations were significantly elevated in pubescent females thirty minutes after they were exposed to the adult male when compared to concentrations in pubescent females that were paired with an adult female. (**B**) Concentrations were elevated two hours later in pubescent females that were paired with an adult male when compared to concentrations in pubescent females that were placed in novel context. (**C**) Learning the classically conditioned eyeblink response was assessed in females exposed to the adult male. Performance during trace conditioning was reduced in those females (SCAR) when compared to females that were not exposed to an adult male (No SCAR). The dotted line indicates the 60% learning criterion that was established as a measure of successful learning of the conditioned response.

The SCAR experience was stressful for the female, as indicated by elevated concentrations of the stress hormone corticosterone, which is released from the adrenal glands during a stressful experience. Concentrations were elevated in the pubescent female 30-min after the first exposure to an adult male when compared to the concentrations that were released when she was placed with an adult female in a novel setting (*t*_(13)_ = 2.59; p < 0.05; Fig. 2A). In a separate experiment, corticosterone concentrations in pubescent females exposed to the adult male for 30-min were elevated two hours later when compared to concentrations in a pubescent female that was left alone in a novel context for 30-min and returned to the home cage (*t*_(9)_ = 3.07, *p* < 0.05; Fig. 2B). These data suggest that social interaction with the opposite sex is more stressful than interaction with the same sex and more stressful than being left alone in a novel context, at least in the pubescent female rodent.

### Experiment 3: Does SCAR disrupt associative learning in the pubescent female?

In a third experiment, we examined the effect of SCAR exposures on learning the classically-conditioned eyeblink response using a trace procedure. Electromyography (EMG) activity from the eyelid was used to assess eyeblink activity through the muscle. Electrodes were implanted around the eyelid to deliver an unconditioned stimulus (US). During surgery, rodents were injected with sodium pentobarbital (35mg/kg), which was supplemented with an isoflurane inhalant. Two pairs of electrodes (insulated stainless steel wire 0.005 in.) were attached to a head stage and implanted through the upper eyelid (orbicularis occuli muscle). Insulation around the wire was removed from a section of each electrode in order to make contact with the muscle. The head stage was positioned using four screws and secured with dental acrylic. After surgery, rats were kept warm and under observation until recovery from anesthesia. Rats were provided Children’s Acetaminophen (conc. 32mg/ml), after surgery at a 112mg/kg dose, administered orally, and allowed at least 2 days recovery prior to training.

At PND 35, a female pubescent rat (n = 6) was exposed to an adult sexually-experienced male for 30-min each day or placed alone (n = 6) in the cage for 30-min. After the fifth SCAR exposure, eyeblink electrode surgery was performed, as described above. After two days of recovery, the females were once again exposed to the adult male each day (SCAR) or left alone in a cage without the male (No SCAR). On the eighth day, each female was exposed to the male for 30-min and then removed from the SCAR exposure and transferred to the conditioning room. The electrodes were connected to the recording equipment and they were acclimated to the training apparatus for one hour. The next day, each female was exposed to the adult male, as before, and then trained with 200 trials of trace conditioning. This procedure was repeated for four days, for a total of 800 trials of training.

A trace conditioning procedure was used, during which the animal is trained to learn the temporal relationship between a white noise conditioned stimulus (CS) and an unconditioned stimulus (US) of periorbital eyelid stimulation. The white noise was delivered at 80 dB for 250 ms, separated by a 500 ms trace interval, and ending with stimulation of the eyelid at 0.5 mA for 100 ms. EMG activity was recorded throughout each trial (excluding the US) to assess and analyze percentage of adaptive eyeblink responses (those that occurred during the trace interval). Eyeblinks in response to the CS were assessed as significant changes in the magnitude and duration from the baseline EMG response. An eyeblink was counted if the EMG activity exceeded 10-ms, 0.3-mV, and was at least three standard deviations (SD) more than the baseline prestimulus EMG response. Those responses that occurred during the 500-ms trace interval and before the US were considered conditioned responses (CRs). As noted, all rats were provided 200 trials each day for 4 consecutive days. Animals that emitted at least 60% conditioned responding in any one session over the course of four days were considered to have learned the CR.

#### Results Experiment 3

A repeated measures ANOVA was conducted using performance on eight blocks of 100 trials as the dependent measures. As expected, the main effect of training was highly significant [*F* (7,70) = 7.89, *p* < 0.001], indicating that the number of CRs increased over blocks and therefore learning occurred. During the first 100 trials, when most of the learning occurs, pubescent females exposed to the adult male emitted fewer CRs than the females that were not exposed to the adult male [F (4,40) = 3.28; p < 0.05]. Females exposed to the adult male (SCAR) also emitted fewer CRs across blocks of 100 trials over the four days of training [F (1,10 = 5.78; p < 0.05; Fig. 2C). These results suggest that both groups learned, but females exposed to the adult male produced fewer well-timed CRs (i.e. during the trace interval). The percentage of CRs was neither increasing on the last day (p = 0.11), suggesting a plateau in learning; yet performances remained different between females exposed to the adult male and those not exposed (p < 0.001). The conditioning data were further analyzed using an arbitrary learning criterion of 60% responding. This criterion is shown as a dotted line in Fig. 2C to indicate 60% conditioned responding. All females in the control group (No SCAR; 6/6) reached a learning criterion of 60% responding with 800 trials, whereas only 50% of females (3/6) in the SCAR group did.

### Experiment 4: Does SCAR disrupt maternal sensitization?

**Figure 3:**
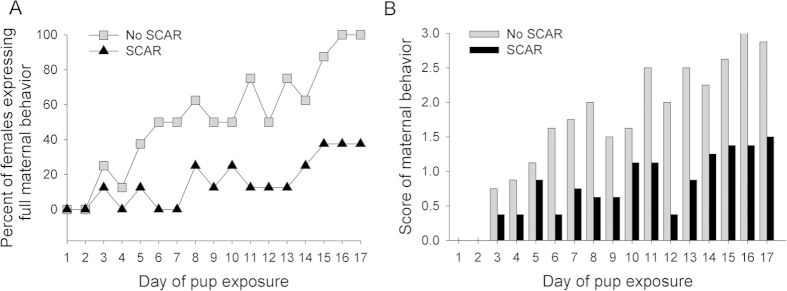
SCAR disrupts maternal behavior and sensitization. (**A**) Pubescent females that were exposed to the adult male during puberty (SCAR) were less likely to learn to express maternal behaviors over the course of 17 days. Only three of these females (3/8) expressed maternal behaviors whereas all of the virgin females that were not exposed to the adult male did (8/8). (**B**) The numbers of maternal behaviors (licking, retrieving and pup grouping) were tallied each day for a potential total score of 3. Pubescent females exposed to the adult male (SCAR) expressed fewer of these behaviors than did females not exposed to the adult male (No SCAR).

Adult virgin females can express maternal behaviors over time in response to newborn pup exposure^14^,^20^ through a process known as maternal sensitization. These same behaviors were expressed by females in puberty, as shown in Fig. 3A. To determine whether the SCAR exposures reduce maternal sensitization, each pubescent virgin female rat (n = 8) was exposed to the adult male (SCAR) for 21 consecutive days beginning on PND35. As a control, a group of pubescent females (n = 8) were each placed alone in an empty cage according to the same schedule. On the fifth day of SCAR exposures, PND39, two newborn postnatal pups (PND 1–10) were placed in the pubescent female’s home cage for 24-h. The pups were born from non-experimental dams and therefore returned to their original dams for nutrition and care every 24 hours, spending 24-h with their lactating dams. Newborn pup health was fair; if pups were neglected by their original dam, they were removed from the study. For maternal behavior observations, pups were place at opposite sides of the home cage, and maternal behaviors were observed and recorded for the first 10 minutes after placement. The recorded behaviors were 1) licking/grooming of pups, 2) retrieving of either one or two pups, and 3) grouping of pups. Once the full complement of maternal behaviors was expressed for two consecutive days, the female was considered to have expressed maternal sensitization.

#### Results Experiment 4

The following maternal behaviors were analyzed: licking, retrieving and grouping of pups. The numbers of maternal behaviors were tallied each day for a potential total score of 3. Repeated-measures analysis of variance across days of exposure to the pups and the SCAR condition indicated a significant increase in maternal behavior [F (16) = 8.39; p < 0.05; Fig. 3B] and an interaction with the SCAR exposures [F (1,16) = 2.18; p < 0.01]. Significant differences between the group behaviors emerged within seven days of pup exposure (p < 0.05). Most of the SCAR females did not express all three maternal behaviors whereas females not exposed to the male (8/8) expressed maternal behaviors, usually within 5–7 days (Fig. 3A).

### Experiment 5. Does SCAR disrupt newly-generated cells in the hippocampus?

First, we assessed the potential impact of SCAR exposures on the number of cells proliferating in the dentate gyrus within the first two hours of a SCAR exposure. Females were injected with one intraperitoneal injection of 5-bromo-2-deoxyuridine (BrdU; 200 mg/kg) immediately before a single 30-min SCAR exposure and sacrificed 2 hours following the BrdU injection (n = 5). Cell numbers were compared to those in a group that were injected with BrdU and sacrificed two hours later (n = 6). Second, we assessed the potential impact of SCAR exposures on the number of cells that were labeled with BrdU after exposure to the adult male over the course of one week. To do this, a group of pubescent females were exposed to the adult male each day for 8 consecutive days beginning at PND35 (n = 7). They were injected with BrdU before the 6^th^ exposure (PND 40) and sacrificed one week after the injection. Another group of females were left alone in their home cages (n = 4), given a BrdU injection on PND 40, and sacrificed one week later. To examine the effects of SCAR on cell survival, a group of animals was injected with BrdU once and sacrificed twenty-one days after the one BrdU injection (No SCAR; n = 7). The number of cells that were labeled with BrdU was compared to the numbers in a group (SCAR; n = 5) that was injected with BrdU and then exposed for 30-min to the adult male each day for 21 days beginning at PND35.

Immunohistochemistry was conducted to analyze the number of BrdU-labeled cells. Animals were deeply anaesthetized with sodium pentobarbital (100 mg/kg; Butler Schein, Indianapolis, IN, USA) and transcardially perfused with 4% paraformaldehyde in 0.1 M phosphate buffer. Brains were extracted and post-fixed in 4% paraformaldehyde at 4 °C for 24–48-h to preserve tissue structure, before being transferred to phosphate buffered saline (PBS). A vibratome was used to cut 40μm coronal sections through the entire rostral-caudal extent of the dentate gyrus in one hemisphere. This is the standard practice in our laboratory, as no hemispheric differences in proliferation have been observed between the left and right dentate gyrus^21^,^22^. Every twelfth slice was mounted onto a superfrost glass slide (Fisher Scientific, Suwane, GA, USA) and allowed to air dry. Once dry, the tissue was stained using standard peroxidase methods to visualize the cells that incorporated BrdU as described previously^22^. Tissue was pretreated with heated 0.1 M citric acid (pH 6.0), rinsed with 0.1 M PBS, incubated in trypsin for 10-min, and denatured in 2N HCl for 30-min with PBS rinses in between. Tissue was incubated overnight in primary mouse anti-BrdU (1: 200; Becton-Dickinson, Franklin Lakes, NJ, USA) and 0.5% Tween-20 (Vector Laboratories, Burlingame, CA, USA). The next day, tissue was rinsed and incubated in biotinylated anti-mouse antibody (1: 200, Vector Laboratories) for 60-min and placed in avidin-biotin-horseradish peroxidase (1: 100; Vectastain ABC Kit, Vector Laboratories) for 60-min. Tissue was placed in diaminobenzidine (DAB SigmaFrost tablets, Sigma Aldrich) for four minutes, rinsed, counterstained with 0.1% cresyl violet, dehydrated, cleared, and cover slipped with Permount glue (Fisher Scientific).

Quantitative microscopic analysis was performed blind to the experimental condition by coding each slide. Estimates of the total number of BrdU-positive cells were determined using a modified unbiased stereology protocol^23^,^24^. Number of BrdU-positive cells in the dentate gyrus of each slice (granule cell layer and hilus) was counted by hand at 1000X on a Nikon Eclipse 80 i light microscope. Ten slices throughout the rostral caudal extent of the hippocampus were collected on the slides and the number was multiplied by 24 to obtain an estimate of the total number of BrdU-positive cells in the dentate gyrus in both hemispheres.

To assess whether maternal “learning” rescued new neurons from death and/or whether SCAR would prevent their survival, groups of pubescent females that were exposed to the adult male (n = 7) or not (No SCAR; n = 7) in the previous experiment were injected once with BrdU and cell numbers were compared to those in additional groups that were not exposed to pups (SCAR, n = 5; No SCAR, n = 7). As noted, one week later, just as most of the new cells would undergo programmed cell death, maternal sensitization with the offspring began. Females were housed each evening with offspring and their maternal behaviors were recorded and analyzed, as described in Experiment 4. Three weeks after the BrdU injection, four groups of females were given a lethal dose of sodium pentobarbital and brains were prepared for immunohistochemistry and microscopic analyses. Due to the nature of BrdU injections, number of animals in these groups was smaller than numbers from data presented in Experiment 4. In addition, we analyzed potential differences in cell numbers between the dorsal and ventral hippocampus. To accomplish this, BrdU labeled cells in the ventral region were compared to those in the dorsal according to interaural coordinates. The dorsal hippocampus was associated with slices from the rostral hippocampus (interaural 3.70 mm to 6.88 mm), whereas the ventral was associated with slices from the caudal hippocampus (interaural 2.28 mm to 3.70 mm), as described^25^.

#### Results Experiment 5

**Figure 4:**
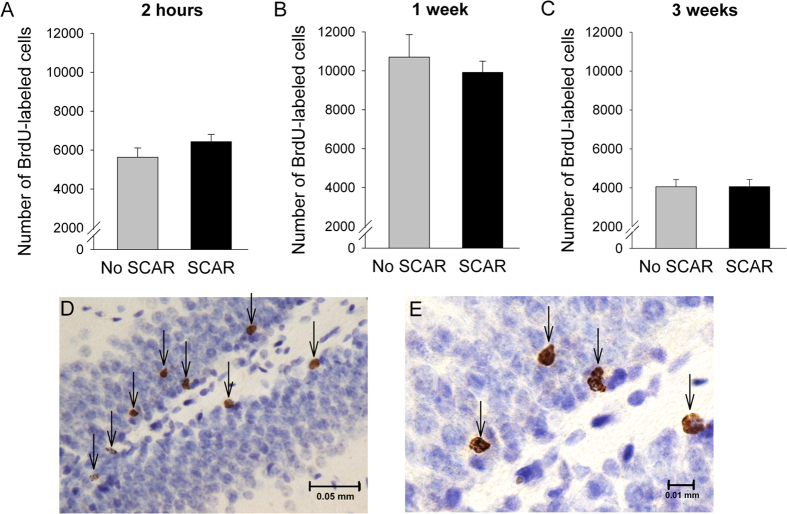
SCAR did not reduce proliferation of newly-generated cells in the hippocampus. (**A**) SCAR exposures did not alter the number of newly-generated (BrdU-labeled) cells two hours later. (**B**) The number of BrdU-labeled cells increased during the week after the BrdU injection but SCAR exposures did not alter the numbers of cells. (**C**) Three weeks later, most BrdU-labeled cells were no longer present and therefore, had presumably died. (**D**,**E**) Representative photomicrographs of BrdU-labeled cells at 400X and 1000X in the dentate gyrus (granule cell layer) of a pubescent female.

**Figure 5:**
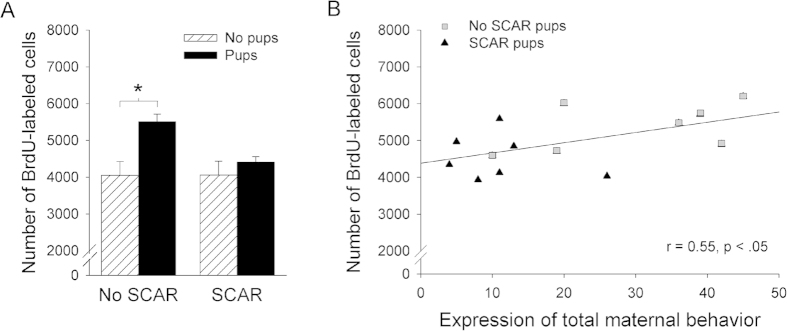
Maternal sensitization and caring of pups related to survival of newly-generated cells in the dentate gyrus. (**A**) Pubescent females that were injected with BrdU and exposed to offspring through the process of maternal sensitization retained more of the BrdU-labeled cells than did pubescent females that were not exposed to the pups. (**B**) Females that were exposed to the adult male were less likely to express maternal behaviors and retained fewer BrdU-labeled cells. Because the vast majority of these cells will mature into neurons, these data suggest that learning to become a mother relates positively to the survival of newly-generated neurons in the female hippocampus.

The numbers of BrdU-labeled cells did not differ between females exposed to the adult male and sacrificed 2-hr or 1-week later (p > 0.05; Fig. 4A,B). We did not observe any differences between the dorsal and ventral hippocampi (p > 0.05) on any of these measures (2 hour, 1 week, 3 weeks). Also, exposure to the adult male alone did not significantly affect the number of surviving BrdU-labeled cells (p = 0.94; Fig. 4C and Fig. 5A). However, the number of BrdU-labeled cells increased in females that had been exposed to pups during maternal sensitization (F_(1,25)_ = 10.03; p < 0.005; Fig. 5A). These data suggest that presence of the pups in the cage may be sufficient to increase the survival of newly-generated neurons in the dentate gyrus of the hippocampus. The interaction between pup exposure and SCAR exposure was nearly significant [F (1,22) = 3.66; p = 0.068). Planned comparisons indicated that the females that were not exposed to adult male but were exposed to pups had more BrdU-labeled cells in the dentate gyrus granule cell layer than females that were not exposed to pups or the adult male (p = 0.002). In contrast, the females that were exposed to the adult male and were exposed to pups did not have significantly more BrdU-labeled cells than those that were not exposed to pups (p = 0.41). There was a significant correlation between the number of cells remaining in the hippocampus at 3 weeks and numbers of maternal behaviors expressed in the presence of the pups (*r* = 0.55; *p* < 0.05). Females that were less likely to express maternal behavior during sensitization retained fewer of the new cells. Therefore, the potential impact of SCAR on the survival of new cells in the hippocampus is not necessarily mediated by the stress of the SCAR experience itself but because it reduced the learning of maternal behavior, which does appear to increase the survival of the newly generated cells. These data are novel for two reasons: first, they indicate that exposure to the offspring may be sufficient to increase the survival of newly-generated cells in the hippocampus. Second, the data suggest that the SCAR experience reduces the survival of newly-generated cells in the female hippocampus through deficits in learning to become maternal.

## Discussion

Sexual aggression and violence is a problem for women and men in many cultures, including the United States. The experience is especially common for young women in puberty and early adulthood. However, sexual aggression is not limited to humans and can occur during sexual behavior and exploration in species ranging from reptiles to rodents to nonhuman primates^26^,^27^,^28^,^29^,^30^,^31^,^32^. It has been hypothesized that aggression, especially physical aggression during sexual exploration, allows the male to gain access to the female for reproductive purposes^27^,^33^,^34^. Many studies have examined aggressive behaviors between males and some have examined aggression between male and females, but most focus on the male response. Very few laboratory models focus exclusively on the female response to sexual aggression, especially those that occur during puberty and early adulthood^35^,^36^,^37^,^38^,^39^. To meet this need, we developed the laboratory model for sexual aggression, known as SCAR, during which a pubescent female is repeatedly exposed to a sexually-experienced adult male conspecific until she reaches young adulthood. During the interaction, the adult male aggressively approaches, pins down and attempts to mount the pubescent female rat even though her vaginal canal is not fully open (Fig. 1). The most consistent behavior recorded was anogenital tracking, whereby the adult male pursues the anogenital region as the female darts around the cage trying to escape. During the interaction, the adult male would oftentimes pin the female down but because she was so small and agile, she was able to flee. There were few if any intromissions, and therefore the interactions did not result in copulation. This is probably because the pubescent female can escape but also because her vaginal canal is not fully open and she is not ovulating. Interestingly, numbers of aggression-related behaviors (pins and anogenital trackings) did not habituate over days and maintained their intensity even after eight days of exposure and as the pubescent female was reaching sexual maturity.

One of the goals of the present set of experiments was to establish SCAR a realistic model of stress in females. From animal laboratory studies, we know that stressful life experience has a multitude of detrimental effects on neuronal and behavioral outcomes. That said, most animal models rely on stressors that are not encountered by humans living in modern society (i.e. restraint stress, aversive shocks or swim stress), much less do they represent stressors commonly experienced by young women. To verify that the encounter with the male was stressful and potentially aversive, we measured corticosterone concentrations, which were enhanced. The mean concentrations were significantly elevated when compared to mean concentrations in a group of pubescent females that were each paired with an adult female (Fig. 2A). In a separate experiment, we determined that the interaction elevated concentrations of corticosterone when compared to a group of females that were each placed in a novel context for the same amount of time (Fig. 2B). Based on these results, we conclude the interaction with the adult male is a stressful experience for the female and more stressful than interacting with another female or exposure to a novel context. Therefore, the SCAR experience is more stressful than novelty, per se. Also, as noted above, the behaviors did not habituate over sessions and remained elevated even eight days later. We did not measure corticosterone concentrations at this time point, but given that the behaviors did not change, it is likely that the corticosterone concentrations would remain elevated. Minimally, these data suggest that the SCAR experience is stressful enough to persistently activate the HPA response over the course of a few hours.

The study of social interaction and aggression has a long history but most studies focus on male/male aggression. One model is similar to ours and referred to as juvenile social subjugation. In these studies, pubescent male or female rodents are placed with adult males for 10-min encounters. Overall, their findings indicated that the female brain was more responsive and less selective in its response to the encounter^39^,^40^. Brain regions that were especially activated include the basolateral nucleus of the amygdala, the bed nucleus of the stria terminalis and the hypothalamus. Cooke and his colleagues also examined depression and anxiety-related behaviors in both sexes after the encounters. Females were especially affected with more time spent in the closed arm of an elevated plus maze, and more helplessness behavior during a forced swim test. We did not measure those behaviors here but would expect similar changes in the pubescent female after daily exposure to the adult male. Instead of measures of depressive behavior, per se, we focused here on processes related to learning. And as shown in Fig. 2C, repeated exposures to the aggressive male during puberty disrupted the female’s ability to learn to associate two stimuli that were separated in time, i.e. during trace conditioning. We also evaluated the effects of SCAR on maternal behaviors related to learning (Fig. 4). Young female rodents will learn to care for offspring, even if they are still virgins. This process of maternal sensitization is often used in animal models to assess changes in maternal behavior and the female brain. Exposure to the aggressive and sexually experienced male disrupted the development and expression of complex maternal behaviors, a response that would limit the number of offspring that survive under naturalistic conditions.

The pubescent brain is especially plastic and vulnerable to stressful life experiences^15^,^41^. The hippocampus generates thousands more cells each day during puberty than adulthood^15^. However, cell production is often decreased by stressful experience. To determine whether the SCAR experience reduces cell proliferation in the hippocampus, groups of pubescent females were exposed to the adult male or not, as before, and then injected with BrdU (a mitosis marker) and sacrificed two hours, one week or three weeks later. This procedure allowed us to evaluate the effects of SCAR (exposure to the adult male) on the proliferation versus the survival of newly-generated cells. Mean numbers of BrdU labeled cells that were present at each of these time points were similar between females exposed to the adult male and those that were not, suggesting that the SCAR experience did not reduce neurogenesis through a decrease in cell proliferation (Fig. 4). As noted, animals in puberty produce many more more newly generated neurons than adult animals^15^. Nonetheless, there was no effect of the SCAR experience on the number of BrdU-labeled cells present 2-h or one week after the initial injection. Rather, the difference occurred three weeks after the initial injection and only in response to the maternal sensitization experience (Fig. 5A). Therefore, the present results indicate a change in the survival of new cells that were already present when the maternal behavior ensued rather than the production of cells, *de novo*.

Even though thousands are born each day, as many as half or even more of the new cells die within just a few weeks of being generated^21^. As shown in Fig. 4, more than half of the new hippocampal cells generated within one week were no longer present within several weeks. In a series of laboratory studies, we have determined that the new cells can be rescued from death by effortful learning, including cells generated during puberty^15^,^16^. We did not examine cell survival in the animals that were trained with trace eyeblink conditioning. However, we would not expect training to rescue new neurons from death in SCAR females, simply because the SCAR females did not learn the conditioned response^42^,^43^,^44^. The present data do indicate that daily interactions with offspring may be sufficient to prevent many of the newly-generated cells from dying in the pubescent females, further suggesting that the presence of offspring can prevent the cell death that normally occurs in these young females. Moreover, newly-generated cells were more likely to survive within the female that learned to express full maternal behaviors. Thus, newly-generated cells in the dentate gyrus of the female hippocampus respond to the experiences of motherhood and therefore, they may play an important role in learning to recognize and take care of offspring. These data are consistent with a previous report indicating that new neurons in adult hippocampi of paternal males respond to interactions with their offspring and may be involved in offspring recognition^45^.

The female brain changes while learning to care for offspring^46^,^47^. As noted in the introduction, exposure to an acute stressful event suppresses associative learning during classical conditioning in the adult female rat. However, stress did not suppress learning in females that are either taking care of offspring naturally (through pregnancy) or through the process of maternal sensitization^14^. Moreover, these effects are relatively permanent to the extent that stress does not suppress this type of learning in females had learned to become maternal at some point in their lives.^48^ A recent study reported that oxytocin administration either systemically or locally in auditory cortex enhanced the retrieval of rat pups by mothers that were not expressing maternal behavior^49^. Based on these data, it is possible that pubescent females exposed to SCAR would learn to express maternal behaviors if they are provided oxytocin ICV^50^ or locally in auditory cortex during maternal sensitization^49^. Such an increase in maternal behaviors should thereby increase the survival of the newly-generated neurons in the dentate gyrus of the hippocampus when compared to similarly-treated females without exposure to oxytocin. Together, these various studies point to neurogenesis as a potential mechanism through which parents come to recognize and learn to care for their young. Therefore, the SCAR model may be useful not only for studying the female response to sexual aggression but also for studying the development of maternal behavior and its potential interaction with neurogenesis in the hippocampus.

## Conclusion

Over thirty percent of women worldwide experience sexual aggression or assault in their lifetime and many of these experiences occur during puberty and young adulthood^51^,^52^. Sexual aggression and trauma is associated with dramatic increases in the incidence of depression and cognitive disruption in women^53^. Moreover, women who have been exposed to severe childhood sexual and/or physical abuse oftentimes suffer from PTSD, which is associated with decreases in amygdala and hippocampal volumes, as well as learning deficits^54^. Furthermore, children of mothers suffering from PTSD are at a greater risk for traumatic experiences, which contribute to their poor developmental prognosis^55^. Despite these and other studies in humans, there is, to our knowledge, no established animal model for evaluating the effects of sexual aggression and trauma in females. The studies reported here present SCAR as a useful model of adolescent sexual trauma in females. This is an important contribution because we know very little about the brain mechanisms which account for the increase in depression and other mood disorders in women who experience sexual trauma and aggression and without an animal model, we are limited in the types of studies that can be conducted. The data presented here further indicate that exposure to SCAR significantly reduces learning and the development of maternal behavior, which has consequences for plasticity in the female brain. We submit that the SCAR model and data that arise from it can be used to develop clinical interventions for girls and young women who have suffered sexual violence and trauma and now must learn to recover^56^,^57^.

## Additional Information

**How to cite this article**: Shors, T. J. *et al.* Sexual Conspecific Aggressive Response (SCAR): A Model of Sexual Trauma that Disrupts Maternal Learning and Plasticity in the Female Brain. *Sci. Rep.* **6**, 18960; doi: 10.1038/srep18960 (2016).
